# Comparing RADseq and microsatellites for estimating genetic diversity and relatedness — Implications for brown trout conservation

**DOI:** 10.1002/ece3.4905

**Published:** 2019-02-06

**Authors:** Alexandre Lemopoulos, Jenni M. Prokkola, Silva Uusi‐Heikkilä, Anti Vasemägi, Ari Huusko, Pekka Hyvärinen, Marja‐Liisa Koljonen, Jarmo Koskiniemi, Anssi Vainikka

**Affiliations:** ^1^ Department of Environmental and Biological Sciences University of Eastern Finland Joensuu Finland; ^2^ Department of Biology University of Turku Turku Finland; ^3^ Institute of Integrative Biology University of Liverpool Liverpool UK; ^4^ Department of Biological and Environmental Science University of Jyväskylä Jyväskylä Finland; ^5^ Department of Aquatic Resources, Institute of Freshwater Research Swedish University of Agricultural Sciences Drottningholm Sweden; ^6^ Estonian University of Life Sciences Institute of Veterinary Medicine and Animal Sciences Tartu Estonia; ^7^ Natural Resources Institute Finland (Luke), Kainuu Fisheries Research Station Paltamo Finland; ^8^ Natural Resources Institute Finland (Luke) Helsinki Finland; ^9^ Department of Agricultural Sciences University of Helsinki Helsinki Finland

**Keywords:** ddRADseq, fisheries, population genetics, relatedness, salmonids

## Abstract

The conservation and management of endangered species requires information on their genetic diversity, relatedness and population structure. The main genetic markers applied for these questions are microsatellites and single nucleotide polymorphisms (SNPs), the latter of which remain the more resource demanding approach in most cases. Here, we compare the performance of two approaches, SNPs obtained by restriction‐site‐associated DNA sequencing (RADseq) and 16 DNA microsatellite loci, for estimating genetic diversity, relatedness and genetic differentiation of three, small, geographically close wild brown trout (*Salmo trutta*) populations and a regionally used hatchery strain. The genetic differentiation, quantified as *F*_ST_, was similar when measured using 16 microsatellites and 4,876 SNPs. Based on both marker types, each brown trout population represented a distinct gene pool with a low level of interbreeding. Analysis of SNPs identified half‐ and full‐siblings with a higher probability than the analysis based on microsatellites, and SNPs outperformed microsatellites in estimating individual‐level multilocus heterozygosity. Overall, the results indicated that moderately polymorphic microsatellites and SNPs from RADseq agreed on estimates of population genetic structure in moderately diverged, small populations, but RADseq outperformed microsatellites for applications that required individual‐level genotype information, such as quantifying relatedness and individual‐level heterozygosity. The results can be applied to other small populations with low or moderate levels of genetic diversity.

## 1 INTRODUCTION

Information on genetic variation within and among populations is paramount in understanding the long‐term impacts of human activities. These may include harvesting (Allendorf & Hard, 2009; Henriques et al., 2016), polluting (Paris, King, & Stevens, 2015), habitat destruction and fragmentation (Balkenhol & Waits, 2009; Keyghobadi, 2007; Wofford, Gresswell, & Banks, 2005), and reintroduction and enhancement of wild populations with releases of human‐reared individuals (Anderson, Faulds, Atlas, & Quinn, 2013; Cochran‐Biederman, Wyman, French, & Loppnow, 2015; Seddon, Armstrong, & Maloney, 2007). Particularly microsatellites, i.e., short repetitive regions in the non‐coding DNA, have been used to analyze genetic differentiation and diversity across populations in different taxa for decades (Guichoux et al., 2011). However, modern sequencing methods are being rapidly developed and replacing widely used methods also in conservation genetics (Narum, Buerkle, Davey, Miller, & Hohenlohe, 2013).

Highly polymorphic microsatellite markers, given that they are available for the target species, can typically resolve the genetic structure of populations reliably even among closely related populations, and provide information on population genetic diversity, average kinship and effective population size (*N_e_*). However, microsatellite markers have limitations such as risk of homoplasy for allele size, the presence of null alleles (Putman & Carbone, 2014; Zhang & Hewitt, 2003) or a potentially insufficient number of polymorphic loci in in the study species, which may limit their resolution power. Single nucleotide polymorphisms (SNPs) obtained by restriction‐site‐associated DNA sequencing (RADseq) producing thousands of loci thus provide an appealing alternative, particularly for species without prior genetic information, or for species and populations known to have limited amount of microsatellite variation because of prior population bottlenecks. Likewise, large panmictic populations, for which the differentiation levels are low, are especially challenging for genetic analysis. Consequently, the RADseq approach has rapidly gained popularity (Andrews, Good, Miller, Luikart, & Hohenlohe, 2016; Davey & Blaxter, 2010).

The advantages of SNP markers over microsatellites include their suitability for comparisons of both strongly and weakly diverged populations, and even species, and in revealing ancestral patterns of genetic structuring compared to microsatellites due to the slower mutation rate of SNPs compared to microsatellite regions (Andrews et al., 2016; Zhang & Hewitt, 2003). In addition, the RADseq approach can provide more reliable inferences on population structure (Bruneaux et al., 2013) and improved resolution for data sets with fewer individuals compared to the microsatellite approach (Jeffries et al., 2016). Likewise, Bradbury et al. (2015) demonstrated that SNPs obtained by RADseq were more accurate than microsatellites for characterizing introgression between Atlantic salmon (*Salmo salar*) from the East and West coasts of the Atlantic Ocean. Despite these advances, more information on wider range of species is still needed to compare the performance and cost‐efficiency of these two marker types in determining population structure especially in small populations in need of conservation actions.

In addition to conservation applications focusing on population‐level metrics, individual‐based metrics, including relatedness, genetic diversity and family structure (full‐sib and half‐sib information) are valuable for managing hatchery breeding strategies, and understanding demographics (Hauser, Baird, Hilborn, Seeb, & Seeb, 2011; Stadele & Vigilant, 2016) and diversity‐fitness‐correlations (Hedrick & Kalinowski, 2000) in wild populations. While the fast mutation rate and high polymorphism of microsatellites allow for resolving fine‐scale population structuring (Putman & Carbone, 2014), they may be less suitable for inferring genome‐wide or individual‐level patterns in genetic diversity (Väli, Einarsson, Waits, & Ellegren, 2008), as the number of sampled loci may not be sufficient to represent the total genome of an individual. It has also been demonstrated that SNPs obtained by RADseq produce more precise estimates of relatedness than microsatellites in a range of bird species (Thrasher, Butcher, Campagna, Webster, & Lovette, 2018). Microsatellites may be less efficient for identifying relatives particularly in populations with prior population bottlenecks and lack of gene flow, which limit allelic diversity. Further, the lack of commonly shared loci across species may be a more serious limitation for the use of microsatellite approach compared to RADseq analysis for, e.g., phylogenetic studies (Eaton & Ree, 2013; Near et al., 2018).

Resolving the relationship between individual‐level genetic diversity (i.e., heterozygosity) and fitness is a long‐standing question in conservation and evolutionary biology: lower diversity is expected to contribute to lower fitness (Hedrick & Kalinowski, 2000). However, the genetic background of populations can influence the observed correlations (e.g., Tiira et al., 2006; Velando, Barros, & Moran, 2015), as can the type of genetic marker used (Miller et al., 2014; Väli et al. 2008). Moreover, published estimates of heterozygosity‐fitness correlation (HFC) often have low correlation coefficients (Chapman, Nakagawa, Coltman, Slate, & Sheldon, 2009). In order to reveal a relevant HFC, the diversity of the applied genetic markers needs to represent individual genetic diversity across large areas of the genome, which is often not true for microsatellite panels (Fischer et al., 2017; Väli et al., 2008) as they usually only represent the most variable loci. Consequently, generally lower HFC has been found using microsatellite loci compared to the more numerous SNP loci; there was an almost five‐fold increase in HFC when measured by RADseq approach in comparison to 10 microsatellite loci in an endangered species with low genetic diversity, the harbour seal (*Phoca vituline*; Hoffman et al., 2014). Furthermore, the minimum number of SNPs required for a reliable estimate of individual heterozygosity can vary between populations (Miller et al., 2014), but there is a lack of studies that have included both several populations and a large numerical range of loci in this evaluation (but see Fischer et al. (2017) for a pool‐Seq approach).

Many salmonids provide excellent examples of systems where geographically connected (even sympatric) populations can be genetically isolated (e.g., Castric, Bonney, & Bernatchez, 2001; Estoup et al., 1998; Vähä, Erkinaro, Niemelä, & Primmer, 2007). Due to tremendous changes in their native breeding habitats, including the construction of dams, and overfishing particularly in the feeding areas, a large number of salmonid populations have become extirpated or declined dramatically (e.g., Bradford & Irvine, 2000; Morita & Yamamoto, 2002). As the stocking strategies (restoration or enhancement releases or both) of hatchery fish to maintain some of the impacted populations continue to be optimized, it remains necessary to characterize the best approaches for evaluating differences between hatchery brood stocks and native stocks. Further, the relatively high costs for DNA sequencing and library preparation in RADseq, as well as the potential challenges of obtaining numerous individuals for genotyping from small populations call for assessments whether there is cost‐efficient increase in resolution to be gained by using RADseq analysis over microsatellites when using a low number of individuals.

In this study, we analyzed the genetic structure and diversity of three wild and one captive‐bred population of brown trout (*Salmo trutta* L.) with both a RADseq approach and a DNA‐microsatellite panel commonly used in brown trout population genetic research (e.g., Debes, Gross, & Vasemägi, 2017; Koljonen, Janatuinen, Saura, & Koskiniemi, 2013, Koljonen, Gross, & Koskiniemi, 2014; Swatdipong, Vasemägi, Niva, Koljonen, & Primmer, 2010). Our applied goal was to support making informed management decisions locally. The main methodological goal was to compare the performance of microsatellite and SNP markers especially in the estimation of individual‐level genetic diversity and relatedness to provide future reference for studies seeking to tailor the methodology to suit a given purpose.

## 2 MATERIAL AND METHODS

### 2.1 Sample collection

**Figure 1:**
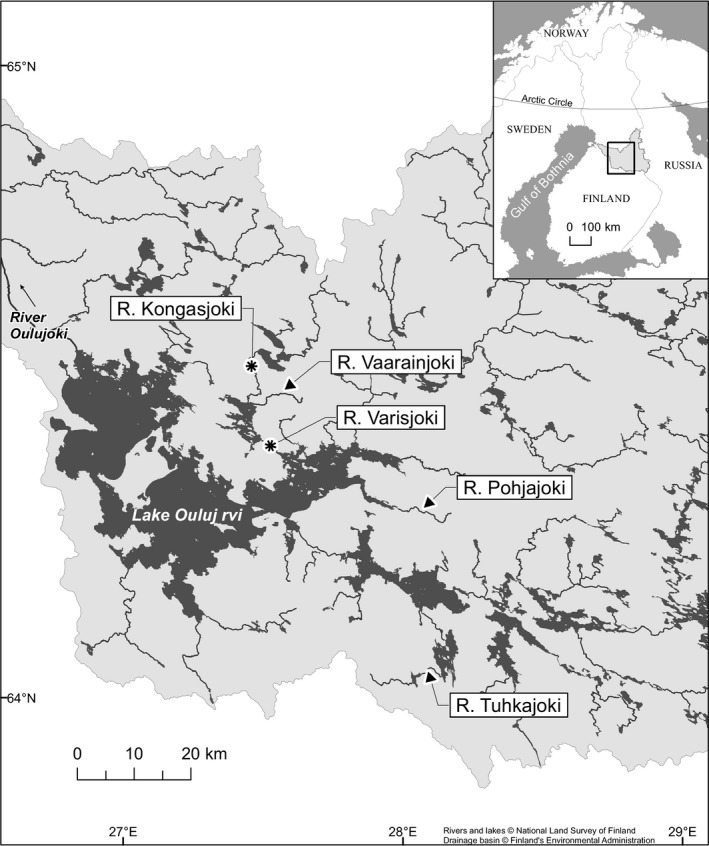
A map showing the River Oulujoki watershed. *Salmo trutta* individuals used in the study were collected from rivers Vaarainjoki, Pohjajoki and Tuhkajoki, indicated by arrows. The rivers that the hatchery stock originated from, Kongasjoki and Varisjoki, are shown with asterisks

Fish sampling and breeding were conducted under a license obtained from the national Animal Experiment board in Finland (license number ESAVI/3443/04.10.07/2015). Wild brown trout were caught in the boreal River Oulujoki watershed from three resident brown trout populations in rivers Tuhkajoki (17 September 2013), Pohjajoki (16–17 September 2015) and Vaarainjoki (28–30 September 2010, 15 September–11 October 2011 and 2 October 2012) using electrofishing and transported to the Kainuu Fisheries Research station (www.kfrs.fi), Paltamo, Finland (Table 1, Figure 1). These populations were assumed to be resident due to the absence of observed migrants in these rivers and unpublished experimental data (personal observation P. Hyvärinen, A. Vainikka). River Pohjajoki fish were sampled under anesthesia immediately after the initial capture. Any mortality prior to sampling was negligible in all populations. Samples from the Tuhkajoki, Vaarainjoki and a hatchery brood stock (details below) were collected at Kainuu Fisheries Research Station by snipping a small piece of caudal fin under anesthesia (benzocaine) during artificial breeding between 12 and 22 October 2015. For each river and the hatchery stock, 30 fish were sampled, for a total of 120 individuals.

**Table 1:** Sampling coordinates and summary of individuals used in the analysis with each marker. For RADseq data, *N* before filtering shown in parentheses

		Microsatellite data		RADseq data	
Population	Lat/Lon	*N*	Sex (F/M/immature)	*N*	Sex (F/M/immature)
Pohjajoki	64^°^ 17′ 50.703″ *N*/28^°^ 3′ 0.416″ E	30	0/4/26	11 (12)	0/4/8
Tuhkajoki	64^°^ 2′ 28.337″ *N*/28^°^ 7′ 10.099″ E	30	6/12/12	9 (11)	3/3/5
Vaarainjoki	64^°^ 28′ 50.510″ *N*/27^°^ 34′ 17.340″ E	30	15/15/0	29	15/14/0
Hatchery stock	Details in text	30	15/15/0	26 (28)	15/13/0

Samples from the hatchery brood stock included in this study (Ouv) were collected from the second generation of this stock. The stock was founded in October 2000 by using adfluvial brown trout from two previous hatchery brood stocks originating from two connected rivers, River Varisjoki and upstream River Kongasjoki (Figure 1). The former was originally founded in 1960–1970s and fish from other wild populations from the same watercourse were possibly mixed into it without reliable records in the 1960–1970s. The latter was founded in the 1970–1980s.

Total DNA was extracted from fin clips preserved in pure ethanol or dried scales using the Omega bio‐tek E.Z.N:A Tissue DNA kit or Macherey‐Nagel NucleoSpin Tissue kit. The quality of total DNA was controlled with electrophoresis on a 1% agarose gel and with fluorometric measurements using Qubit 2.0 with Qubit^®^ dsDNA HS Assay Kit (ThermoFisher Scientific).

### 2.2 Microsatellite analysis

Allelic variation was determined at 16 microsatellite loci (Supporting information Table S1) for all 120 individuals (Table 1). For each sample, two multiplex PCR reactions were performed using the Qiagen Type‐it Microsatellite kit in a 10‐μl reaction volume with 3 μl of extracted DNA, 5 μl of kit master mix and primers with concentrations and dyes as presented in Supporting information. PCR reactions were carried out in PTC200 Thermal Cyclers (MJ Research), and the temperature profile of the PCR program was suggested in the Type‐it Microsatellite kit manual. The annealing temperature was 56°C. The amplification products were separated by capillary electrophoresis on AB3130 Genetic Analyzers (Applied Biosystems, Foster City, CA). The sizes of the microsatellite alleles were determined using Genemapper v. 4.0 software (Applied Biosystems, Foster City, CA), and manually checked. Deviations from Hardy‐Weinberg equilibrium in each population were tested using function *hw.test* from pegas package (Paradis, 2010) in R environment. A few loci showed a significant deviation from equilibrium within populations (2 in Tuhkajoki, 1 in Pohjajoki, 4 in Vaarainjoki and 2 in hatchery stock). However, as all microsatellite loci were in equilibrium in at least two populations, none were excluded from analysis.

### 2.3 Sequencing, genotyping and SNP calling

The sequencing libraries were prepared using samples outlined in Table 1 and Supporting information Table S2. From each sample, 100 ng of genomic DNA along with PstI‐HF (5′CTGCAG 3′) and BamHI‐HF (5′GGATCC 3′) restriction enzymes was used. The protocol used was the same as in Lemopoulos, Uusi‐Heikkilä, Vasemägi et al. (2018a). Briefly, individual barcodes were ligated to the forward ends before pooling the individuals into a single library. The library was purified with PCR purification and size selected into 280–320 bp fragments on an E‐Gel Size select 2% Agarose gel (Invitrogen, CA, USA). Amplification through PCR was then performed and the product was purified using SPRI‐Beads, removing fragments <100 bp. The DNA concentration of the libraries was quantified using Qubit 2.0. Size and quantity of contained fragments were assessed with Agilent 2,100 Bioanalyzer (Agilent Technologies, CA, USA). Samples were pooled into one sequencing lane. Four samples were re‐sequenced on different sequencing lanes to determine the genotyping error rate. Libraries were sequenced on Illumina HiSeq 2,500 with the rapid run option by a commercial service provider, Turku Centre for Biotechnology (BTK), in Turku, Finland.

A total of 113,885,773 reads were retained after quality filtering (Supporting information Table S2), as well as 6.08 million reads used for estimating sequencing error rate, which was 5% at the SNP level (including the error rate from contig assembly and SNP calling). The average coverage depth per individual after filtering was 42.64x. The obtained RAD‐data were analyzed using Stacks, v. 1.40 (Catchen, Hohenlohe, Bassman, Amores, & Cresko, 2013). *Process_Radtags* function was used for demultiplexing, quality filtering (q) and cleaning (‐c). Orthologous tags were assembled, catalogued and matched using *denovo* pipeline, in which the optimal parameters were obtained following Paris, Stevens, and Catchen (2017). Minimum coverage (‐m), maximum mismatches between loci for a single individual (‐M) and the maximum mismatches (‐n) between loci for catalogue building were all set to 2. All other parameters were set to default. *Population* function was run for the SNP calling. On the first call, only loci that were present in at least 50% of all the individuals were kept while the rest of the parameters were set to defaults. Further filtering was done in R using the *stackr* (Gosselin & Bernatchez, 2016) and *grur* (Gosselin, 2017) packages. To exclude uninformative markers and samples with too much missing data, the number of populations and individuals where a locus had to be present were assessed based on the data (*missing_visualisation *function), after which new parameters were again passed onto STACKS’ *population* function. Finally, only loci present in all four populations (‐p) and in 60% of the individuals (‐r) were retained. Based on this dataset, a total of five individuals with more than 20% of missing data (Table 1), and markers with more than 30% of missing data were discarded (using *stackr*). This was done in order to remove potential sequencing errors and uninformative missing data that could potentially bias the results (see *stackr* package guidelines). In addition, a filter for marker heterozygosity (maximum threshold: 0.5, as in Hohenlohe, Amish, Catchen, Allendorf, and Luikart (2011)) was applied to remove potential sequencing errors. Individual heterozygosity was between 0.14 and 0.22; thus, no individuals were excluded based on heterozygosity. Markers were further filtered for minor allele frequency based on a local (0.02) and a global (0.005) threshold. This dataset was then assessed using *missing_*visualisation function and identity‐by‐missingness analysis with *grur* to confirm that populations did not cluster based on missing data. Further, only loci that were under Hardy‐Weinberg equilibrium as defined by *p‐*value threshold >0.05 in at least two populations were retained (HW tests made using *pegas* package; Paradis, 2010 in R).

Because the sex ratio of the samples from two of the studied populations was not known, the SNP dataset was checked for the presence of sex‐linked markers to avoid introducing bias into the analysis (Benestan et al., 2017). Genetic variation was compared in all individuals with known sex using BAYESCAN v2.01 (31 females and 32 males; Foll & Gaggiotti, 2008) to detect potential sex‐linked outliers. The input file was created using PGD software v2.1.0.3 (Lischer & Excoffier, 2012) and all default parameters were used in Bayescan. No sex‐linked outlier loci (alpha = 0.1) were found in the final set of SNPs, likely indicating that these loci were excluded in the other filtering steps or had low coverage. SNPs were not filtered based on neutrality, as the number of loci under selection in the dataset as a whole is expected to be small (see, e.g., outlier analysis on brown trout in Lemopoulos, Uusi‐Heikkilä, Huusko, Vasemägi, & Vainikka, 2018b). The final RADseq dataset comprised 4,876 loci and 75 individuals. Trimmed read counts in these samples ranged from 148,214 to 3,116,841 (Supporting information Table S2).

### 2.4 Genetic diversity and differentiation between populations

The following analyses were conducted on three datasets: microsatellite data from all individuals, microsatellite data from the same individuals as in the final RADseq data, and the SNPs from the final RADseq data.

Pairwise and global *F*_ST_ – values (Nei, 1973) were analyzed using package *hierfstat* (Goudet & Jombart, 2015). Total allele counts and allelic richness were measured using package *PopGenReport* v.3.0, and expected heterozygosity (*H*_e_) using package *adegenet* v.2.0.1 (Jombart, 2008; Jombart & Ahmed, 2011) in R. *P*‐values for pairwise *F*_ST_ – values were obtained using 999 MCMC permutations. Effective population size (*N*_e_) was calculated using the method based on the linkage disequilibrium (LD; Hill, 1981; Waples, 2006; Waples & Do, 2010) as implemented in NeEstimator v.2.1 (Do et al., 2014). Minimum allele frequency of 0.02 and non‐parametric jackknifed confidence intervals were used for all analyses of *N*_e_ (Jones, Ovenden, & Wang, 2016). The estimates of *N*_e_ obtained for SNP data are known to suffer from a downward bias and were thus corrected using the equation 1a from Waples, Larson, and Waples (2016) (haploid chromosome number = 40; Leitwein et al., 2017). The corrected *N*_e_ was then calculated as: *N*_e_ from NeEstimator/0.906.

### 2.5 Multivariate analysis and bayesian clustering

The clustering of samples into four populations was visualized using Discriminant Analysis for Principal Components (DAPC) from package *adegenet* v. 2.0.1 (Jombart, 2008; Jombart & Ahmed, 2011) in R. The number of principal components retained for DAPC was obtained by cross‐validation, selecting the lowest number of components where the correct assignment probability levelled (13 for SNPs, 4 for both datasets of microsatellites).

The likely number of distinct source populations and admixture between populations were additionally analyzed using STRUCTURE (Pritchard, Stephens, & Donnelly, 2000). STRUCTURE was repeated 20 times using a burn‐in of 50,000 followed by 100,000 iterations for microsatellites and a burn‐in of 200,000 and 200,000 iterations for the RADseq dataset, for each *K* value 2–7, after which the optimal *K* value was determined using CLUMPAK (Kopelman, Mayzel, Jakobsson, Rosenberg, & Mayrose, 2015), selecting the *K* where the mean ln likelihood converged. Once the optimal *K* (4) was determined, the 20 runs of STRUCTURE using *K* = 4 were combined using LargeKGreedy algorithm in CLUMPP v.1.1.2 (Jakobsson & Rosenberg, 2007), to which the input file was generated using STRUCTURE HARVESTER (Earl & Vonholdt, 2012). The output file of CLUMPP was visualized using DISTRUCT v.1.1 (Rosenberg, 2004). The SNP and STRUCTURE analyses were conducted using the CSC – IT Center for Science Ltd clusters in Finland.

### 2.6 Family structure and relatedness

Family structure within populations was assessed in the individuals with both SNP and microsatellite data available using COLONY v. 2.0.6.2 (Jones & Wang, 2010) with random mating model (Wang, 2016). The error rate was set at 0.05 for SNPs (based on repeated genotyping of five individuals) and 0.0001 for microsatellites (based on empirical, unpublished data). Each analysis was run twice using medium run length to confirm that identical results were obtained on both runs. Because the removal of siblings from population genetic datasets can lead to a loss of information on genetic divergence between populations (Waples & Anderson, 2017), all samples were included in the analyses of population structure regardless of family origin. Relatedness was calculated based on the number of alleles shared between individuals (*B_xy_*; Li & Horvitz, 1953). Differences in mean pairwise relatedness based on 1,000 bootstrapped replicates of random subsets of loci (1–16 loci for microsatellites and 1–100 loci for SNPs) were obtained using function *loci.test* from package *demerelate* in R.

### 2.7 Individual multilocus heterozygosities

Standardized multilocus heterozygosities (sMLH), i.e., the relationship between the heterozygous loci for an individual and the sum of observed average heterozygosity in the population (Coltman, Pilkington, Smith, & Pemberton, 1999), were measured to compare if microsatellite and SNP markers from the same individuals provide comparable diversity estimates at individual level. sMLH values were obtained using the *inbreedR* package (Stoffel et al., 2016). Pearson correlation was used to evaluate the similarity of the sMLH estimates between microsatellite and SNP markers. In addition, to test how reliably sMLH could be estimated using different numbers of markers, the correlation between two equal‐sized, random subsets of markers (i.e., heterozygosity‐heterozygosity correlation; Balloux, Amos, & Coulson, 2004) was calculated across all individuals and within populations (repeated for 1,000 times). The strength of the correlation between sMLH values from the same‐size subsets of markers indicates the preciseness of the estimates. Subsets of eight microsatellites and 100/500/1,000/1,500/2,000 SNPs were evaluated. Violin plots of the results were produced using *ggplot2* (Wickham, 2009) and *easyggplot2* (Kassambara, 2014). R version v. 3.3.2 (R Core Team 2016) was used throughout the analyses.

## 3 RESULTS

### 3.1 Explorative multivariate analysis

**Figure 2:**
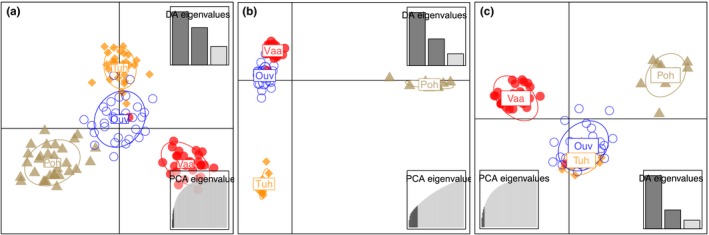
Grouping of *Salmo trutta* samples on DAPC based on full microsatellite data (a), RADseq data (b) and microsatellite data (c) from the same individuals. Populations indicated by labelled symbols, hatchery stock (Ouv), Pohjajoki (Poh), Tuhkajoki (Tuh), Vaarainjoki (Vaa)

Using DAPC, each population was separated from the others based on both microsatellites (Figure 2a) and SNPs (Figure 2b). The hatchery stock (Ouv) and River Tuhkajoki population clustered together using microsatellite data from individuals included in RADseq (Figure 2c), thus displaying only three groups in total.

### 3.2 Bayesian clustering using structure

**Figure 3:**
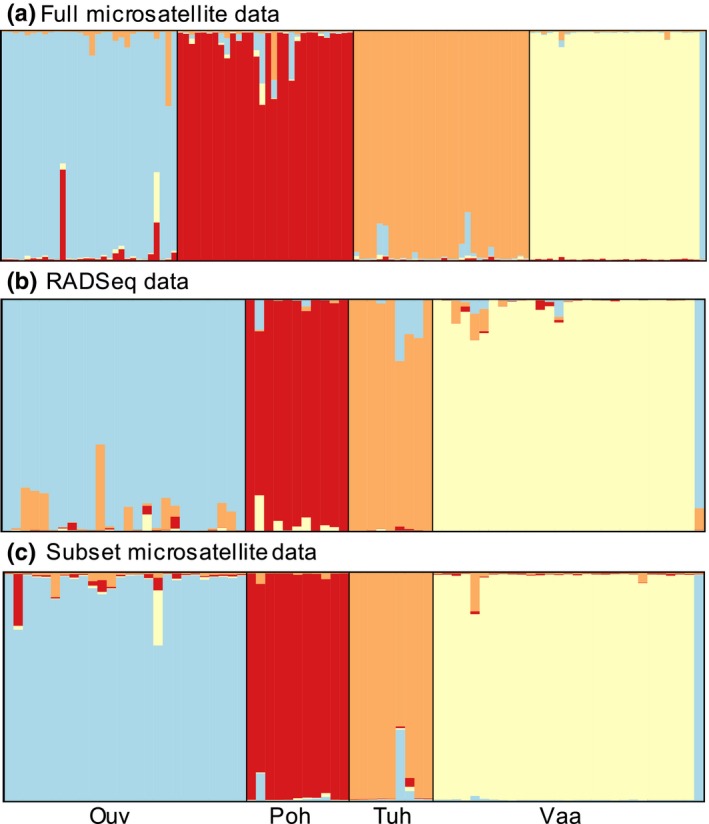
DISTRUCT plots showing posterior probabilities of *Salmo trutta* individual genotypes (as bars) assigned to each population based on microsatellite data from all 120 individuals (a), RADseq data from 75 individuals (b), and microsatellite data from the same 75 individuals (c). The expected populations are separated by black lines. Populations from left to right hatchery stock (Ouv), Pohjajoki (Poh), Tuhkajoki (Tuh), Vaarainjoki (Vaa)

The average likelihood of 20 independent STRUCTURE runs converged at *K* = 4 with all three datasets. There was low admixture between populations (Figure 3a‐c). Both DAPC and STRUCTURE analysis grouped one individual from Vaarainjoki population with the hatchery fish in all datasets (showing that the methods assigned population of origin equally well; Figure 3a–c). In further analyses, this individual was kept in the Vaarainjoki population, as it was originally captured in the river, although it may have been bred in the hatchery.

### 3.3 Genetic diversity and differentiation

In total, 3–18 alleles were found in the 16 microsatellite loci (the sum of all alleles was 143), with a mean of 4–6 alleles across loci within populations (Table 2). Nearly half (48%) of the loci had at most four alleles within populations. 4,876 SNPs were included in the final dataset obtained by RADseq. Both microsatellite and SNP markers identified the hatchery population as genetically more diverse than the wild populations based on *H*_e_ and allelic richness (Table 2). Two individuals from the Vaarainjoki population shared an identical multilocus genotype based on 16 microsatellite markers, which is highly unusual, but may be explained by the low allelic diversity in most of the loci in the population. These individuals were full‐sibs based on SNPs, but in the final set of loci their difference was <2%, which falls within the margin for genotyping error and might indicate that DNA was accidentally collected twice from the same individual, although we consider this unlikely. The average allelic richness was over three times higher for microsatellites than for SNPs (Table 2). The estimates of *N*_e_ were overall low (Table 2). The confidence intervals for *N*_e_ were overlapping and therefore did not indicate significant differences in the estimates across the datasets.

**Table 2:** Genetic diversity and effective population size (*N*_e_) in all studied populations across the three datasets. Total number of alleles, mean locus‐specific allelic richness (Ar mean) and the estimates of expected heterozygosity (*H*_e_) and *N*_e_ shown as measured from microsatellites in 120 individuals (A) and RADseq (B) and microsatellites (C) on the same 75 individuals. LD‐based estimates of *N*_e_ are shown with 95% non‐parametric jackknifed confidence intervals. Note the difference in scale for *H*_e_: the theoretical maximum is 1 for multiallelic microsatellites and 0.5 for bi‐allelic SNPs

	A				B				C			
	Allele count	Ar mean	*H*_e_	*N*_e_	Allele count	Ar mean	*H*_e_	*N*_e_	Allele count	Ar mean	*H*_e_	*N*_e_
Pohjajoki	68	4.01	0.53	14.8 (6.9–34.7)	6,566	1.26	0.09	13.0 (6.3–28.3)	46	2.75	0.48	9.7 (2.8–73)
Tuhkajoki	82	4.89	0.61	22.0 (12.4–46.3)	6,854	1.31	0.1	2.10 (2.9–27.4)	61	3.61	0.58	10.1 (4.4–28.8)
Vaarainjoki	80	4.78	0.59	32.8 (11.5–infinite)	7,555	1.47	0.11	24.5 (12.3–123.8)	80	4.75	0.59	28.8 (10.1–infinite)
Hatchery stock	108	6.31	0.66	53.0 (30.8–133)	8,380	1.61	0.14	96.5 (60.8–149.0)	102	6.042	0.66	57.1 (31.2–316.8)

Based on the complete microsatellite data, the global estimate of *F*_ST _was 0.209 across populations, and was very similar to that based on SNPs (0.211). The pairwise *F*_ST_ values between the three rivers and the hatchery stock were highly significant, and approximately at the same level in all three datasets (Table 3).

**Table 3:** Pairwise *F*_ST_ values for three wild brown trout river populations and one hatchery stock obtained using the full microsatellite dataset (A), and RADseq (B) and microsatellite data (C) on the same individuals

	A			B			C		
	Pohjajoki	Tuhkajoki	Vaarainjoki	Pohjajoki	Tuhkajoki	Vaarainjoki	Pohjajoki	Tuhkajoki	Vaarainjoki
*F*_ST_									
Tuhkajoki	0.154			0.202			0.197		
Vaarainjoki	0.150	0.138		0.134	0.111		0.141	0.116	
Hatchery	0.120	0.074	0.112	0.119	0.064	0.109	0.110	0.067	0.110

### 3.4 Family structure and relatedness

**Figure 4:**
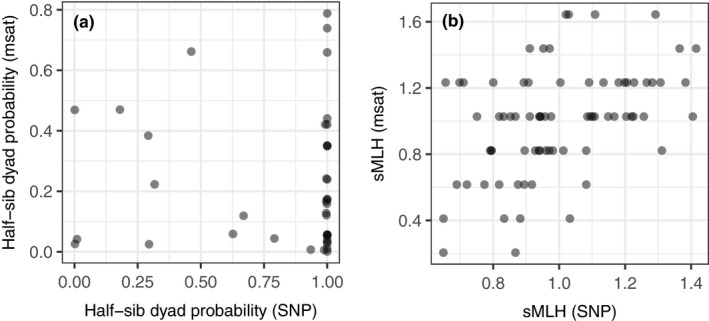
Scatter plots showing *Salmo trutta* half‐sib assignment probabilities across 39 dyads identified with both SNP and microsatellite markers (a) and individual sMLH values for 75 individuals from both markers (b). Results from the whole dataset of SNPs shown in *x*‐axis and from 16 microsatellites in the y‐axis

According to COLONY output, the estimated number of full‐sib families was similar using microsatellite and SNP markers on the same individuals, when empirically determined error rates (0.0001 and 0.05, respectively) were used for each marker (Table 4). In Tuhkajoki, both markers identified largely the same full‐sib families apart from one family identified only with SNPs. In Pohjajoki and Vaarainjoki, none of the identified full‐sib pairs matched except for one pair in Vaarainjoki. In the hatchery stock, microsatellites identified one potential pair of full siblings (but with only 41% exclusion probability) that was classified as neither full nor half‐sibs based on SNPs. The average exclusion probabilities of full‐sib family identification were clearly higher based on SNPs than microsatellites (Table 4), indicating SNPs contained more information to determine sib‐ship. The differences between the markers were even more pronounced for half‐sib assignment, where microsatellite data yielded four times more half‐sib dyads compared to SNP data, with in total 76 based on SNPs and 314 based on microsatellites (Table 4). There was no correlation between the probabilities of the half‐sib dyads that were identified with both markers (Figure 4a, Pearson *r* = −0.04, *N* = 39, *p* = 0.806).

**Table 4:** The number of identified full‐sib families with average exclusion probabilities and half‐sib dyads with average probabilities from microsatellite and SNP data on the same individuals (COLONY software). A comparison of the probabilities of matching half‐sib dyads is shown in Figure 4a

		Pohjajoki	Tuhkajoki	Vaarainjoki	Hatchery stock
Full‐sib families	SNPs	8/0.98	5/0.99	25/1.00	26/0.97
	Microsatellites	10/0.44	4/0.52	26/0.52	25/0.66
Half‐sib dyads	SNPs	9/0.56	3/0.996	34/0.85	30/0.72
	Microsatellites	66/0.18	52/0.07	334/0.18	176/0.26

**Figure 5:**
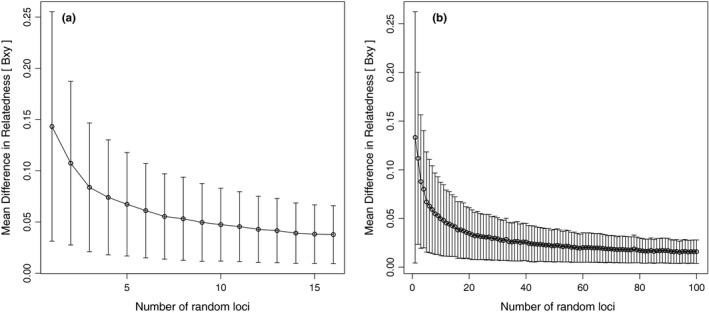
Pairwise differences in relatedness using subsets of loci from RADseq (a) or microsatellite data (b) from the same 75 *Salmo trutta* individuals. Pairwise relatedness between individuals was compared between each subset and the maximum number of loci used (100 SNP or 16 microsatellite loci)

The mean difference in relatedness was low and stable between *ca*. 80–100 SNP loci (Figure 5b). In contrast, when looking at the microsatellite data (Figure 5a), the mean difference in relatedness compared between 1 and 16 loci was overall higher than that measured by SNPs (Figure 5b).

### 3.5 Estimating individual heterozygosity and its accuracy

**Figure 6:**
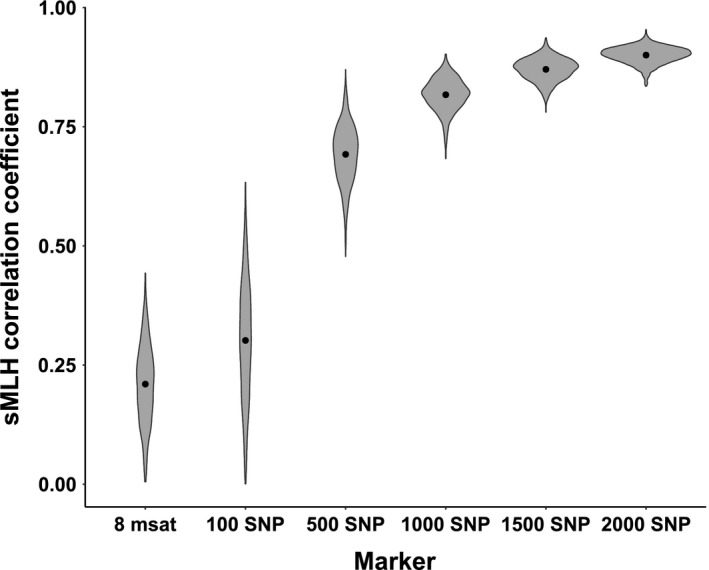
Violin plots showing correlations between subsets of sMLH values in *Salmo trutta* according to different markers. For both microsatellites (msats) and SNPs, the correlation between two equal‐size randomized subsets was calculated for 1,000 replicated sets of loci. Points showing means within each subset

There was a positive, but relatively moderate correlation, between sMLH measured using 16 microsatellites and 4,876 SNPs (Figure 4b, Pearson *r* = 0.45, *t* = 4.29, *df* = 73, *p < *0.001). The range of sMLH was much higher for microsatellites (Figure 4b) for which the average within‐population standard deviation was 2.5 times higher than for the SNP markers. Further, analysis on subsets of SNP markers revealed that the individual genetic diversity was most precisely measured with 1,500 or more SNPs (mean Pearson *r* = 0.87 for 1,500 SNP subset, *r = *0.90 for 2,000 SNP subset, Figure 6). There were no clear differences between populations in the number of SNPs required for a high correlation, but Tuhkajoki and Pohjajoki had higher variation in correlations (based on only 9 and 11 samples; Supporting information Figure S1).

## 4 DISCUSSION

We conducted a comprehensive evaluation of genetic divergence and diversity, and family structure in small, freshwater, postglacial populations of brown trout using both SNP and microsatellite markers. The results first showed that a moderately diverse microsatellite panel of 16 loci covering a total of 147 alleles can produce similar results as >4,800 SNPs obtained by RADseq for quantifying population divergence, and second, that the resolution of SNPs is higher compared to microsatellites in a multivariate analysis. Third, the results suggest that thousands of SNP markers are needed to reliably estimate individual‐level heterozygosity.

### 4.1 Performance of markers at population and individual levels

Moderate and high population divergence was equally well reflected by the two marker types, supporting work in other species. For instance, in the Atlantic salmon, as few as nine SNP markers produced *F*_ST_ values that were correlated to those measured using 14 microsatellites (Ryynänen, Tonteri, Vasemägi, & Primmer, 2007). Other studies have also reported very similar estimates of population divergence between less than a dozen microsatellite loci and >1,000 SNPs in European honeybees (*Apis mellifera mellifera*; Muñoz et al., 2017), *Ar* *abidopsis halleri *(Fischer et al., 2017) and round whitefish (*Prosopium cylindraceum*; Morgan et al., 2017). However, in Crucian carp (*Carassius carassius*), a greater isolation‐by‐distance was identified by RADseq than by microsatellites across Northern Europe (Jeffries et al., 2016). As in Jeffries et al. (2016), we found stronger divergence between populations using the RADseq data in DAPC when comparing the two marker types from the same individuals. A recent population genetics study on mud crab (*Rhithropanopeus harrisii*) also concluded that analyzing even a few individuals from each population with RADseq can be highly informative (Forsström, Ahmad, & Vasemägi, 2017). Alternatives to RADseq‐based approach include, for instance, reduced sets of SNP loci, which can perform nearly as well as thousands of loci in highly diverged populations (Henriques et al., 2018). In addition, increased resolution can be achieved by sequencing a large number of microsatellites first identified with a whole genome sequence scan (Bradbury et al., 2018).

Our results imply that one of the major advantages of RADseq over microsatellite analysis lies in the power to detect family structure within a population. This was shown by much higher probabilities of identified full‐sib and half‐sib families. The lower probabilities obtained with the microsatellite loci can be explained by the small *N*_e_ in the studied populations. In addition, we found only ≤4 alleles in approximately half of the microsatellite loci within populations indicating relatively low diversity dominated by few alleles. Similar allelic richness as in our study have been described also in other brown trout populations from the wild and from hatcheries using partly the same loci (Aho, Rönn, Piironen, & Björklund, 2006; Koljonen et al., 2013; Swatdipong et al., 2010). Low within‐population diversity could be a general phenomenon in endangered populations, suggesting that the results can be applied to other species.

Despite the higher probabilities compared to microsatellite markers, half‐sib identification using SNPs could be confounded by genotyping errors, but little research has been done to investigate this thus far. However, a previous study comparing a custom‐made SNP panel to 10 microsatellite loci for family identification in brown trout found relatively low overlap in full‐sib identification between the markers, and more repeatable results when using *ca*. 3,800 SNPs than when using microsatellites (Linlokken, Haugen, Mathew, Johansen, & Lien, 2016). In contrast, 14 microsatellites and 1,728 SNPs agreed on 98% of full‐sibs identified from 255 individuals in brown trout from River Altja (Ahmad, Debes, Palomar, & Vasemägi, 2018; Debes et al., 2017). The different results on family identification with the two marker types can therefore be partly explained by sample size; in this study, <30 individuals from each population were genotyped using both markers, and Linlokken et al. (2016) used 47–48 individuals per population from three populations. In addition, the frequency and diversity of alleles in each population finally determines the ability of microsatellites to correctly identify family structure. Notably, the use of microhaplotype markers, i.e., markers consisting of regions carrying several SNPs, has emerged as an advantageous approach for population genetic studies and particularly relatedness inference (Baetscher, Clemento, Ng, Anderson, & Garza, 2018). Such approach can be performed with RADSeq data and it has already been used in salmonids, e.g., for Chinook salmon stock identification (McKinney, Seeb, & Seeb, 2017).

Accurate quantification of genetic diversity at the individual level is crucial for HFC studies, which are comparing genetic diversity at specific loci or at the genome‐wide level to variation in fitness‐related traits (Chapman et al., 2009). HFC studies evaluate if the decreased genetic diversity (i.e., inbreeding depression) can reduce fitness in wild populations or make them more vulnerable to environmental disturbances due to lower adaptability to novel challenges (Hedrick & Kalinowski, 2000; Willi, Buskirk, & Hoffmann, 2006). Before SNP markers became widely available, HFC studies typically evaluated links between fitness and at heterozygosity at single or few highly variable loci (Balloux et al., 2004). With the increasing use of RADseq and other SNP‐based genotyping tools, more empirical studies on wider range of species are needed to evaluate the number of markers required to reliably quantify genome‐wide patterns of diversity. Recently, Hoffman et al. (2014) measured correlations in sMLH between subsets of SNP loci similar to this study and found that high accuracy was achieved using 2,000 or more loci. On the other hand, Miller et al. (2014) showed differences in the number of markers required for precise sMLH between two populations of the bighorn sheep (*Ovis canadensis*) using 20–412 SNPs and 5–100 microsatellites: in one population, 20 microsatellites performed as well as 75 SNPs, but in a different population more loci would have been needed from both markers. In our study, we did not find clear differences in the number of SNPs required for precise estimates of sMLH between populations. The lack of population‐specific differences in sMLH preciseness in our study suggests that the population effects in Miller et al. (2014) might be due to a lower number of SNPs used, or differences in overall genetic diversity between populations. Moreover, in *A. halleri*, increasing the number of SNPs beyond 4,000 up to 300,000 led to an increase in the accuracy of individual genome‐wide heterozygosity (Fischer et al., 2017). While the most precise SNP subsets (>1,500 loci) in our study reached ~0.9 heterozygosity‐heterozygosity correlations, perfectly evaluating the accuracy (i.e., a correlation of 1.0 across all loci) of the sMLH estimates would, however, require whole genome data. Overall, both our results and published work indicates that thousands of SNPs are needed to provide accurate estimates of genome‐wide diversity, while estimates based on a few hundred or less SNP markers should be used cautiously.

### 4.2 Implications for conservation and management of brown trout

All the rivers from which the study populations originated have undergone some alterations in the environment due to logging, dams, mining or forestry‐induced decrease in water quality. These effects combined with historically high fishing pressure have further depressed the number of spawning individuals in the study populations. This explains the low genetic diversity and the *N*_e_ estimates that were below 25 according to SNPs. In previous studies on other focal systems, estimates of *N*_e_ below 50 have often been observed, which are in line with our observations (Linlokken et al., 2016; Linlokken, Johansen, & Wilson, 2014; Sonstebo, Borgstrom, & Heun, 2007; Vøllestad, 2017) suggesting that many brown trout populations in brooks are naturally small and strongly genetically differentiated. Each of the studied wild populations appeared to be an isolated unit with very limited gene flow from the other rivers or from the hatchery‐reared fish, which is in line with their historical and present‐day connectivity. Although Pohjajoki and Vaarainjoki are both connected to Lake Oulujärvi, Vaarainjoki first discharges to Lake Kivesjärvi, which is connected to Oulujärvi through River Varisjoki. It is thus unlikely that resident brown trout would make a journey from Vaarainjoki to Pohjajoki, or *vice versa*. On the other hand, the resident Tuhkajoki population has been separated from the other populations by a natural migration barrier (nowadays also by multiple dams), thus explaining its divergence from the other wild populations. Overall, differences among populations can also be partly explained by founder effects, as they may have been initially formed by few individuals after the last glacial period since *ca*. 10,000 years, as well as by genetic drift, partly due to their resident life‐history strategy and small population size even in fully natural conditions. The RADseq approach applied here was also used to assess the population genetic structure of brown trout in River Koutajoki watershed in Finland (Lemopoulos, Uusi‐Heikkilä, Vasemägi et al., 2018a), where the pairwise *F*_ST_ values were in a similar range as in the River Oulujoki watershed. These values are comparable to other brown trout populations with migration barriers (0.099 ± 0.005), albeit higher than those of populations without migration barriers (0.043 ± 0.003; Koljonen et al., 2013; values from Vøllestad, 2017).

Both marker types evidenced that stocked fish can disperse to the stream habitats of resident trout, as one individual of identified hatchery origin was caught in Vaarainjoki during spawning time. Hatchery fish are generally stocked directly to Lake Oulujärvi, the close‐by Lake Kivesjärvi, or to rivers Varisjoki or Kongasjoki, as 2‐ or 3‐year‐old smolts, and can thereafter disperse to Vaarainjoki. Small‐scale introductions may have been made also in Tuhkajoki, which could explain the presence of hatchery genotypes in this population. Several studies have shown that introgression from hatchery stocks to wild fish populations occurs especially when the number of stocked fish is high compared to the number of wild individuals (Hansen, 2002; Hindar, Ryman, & Utter, 1991; Ozerov et al., 2016; Salminen, Koljonen, Säisä, & Ruuhijarvi, 2012), as is the case in Lake Oulujärvi area. Further, introgression from hatchery‐reared fish can occur at sites distantly located to the stocking location (Vasemägi, Gross, Paaver, Koljonen, & Nilsson, 2005; Finnegan & Stevens 2008). Wild brown trout populations may be locally adapted to their native environments (Jensen et al., 2008; Westley, Ward, & Fleming, 2013) and introductions of genetically differentiated hatchery‐reared fish could negatively impact such adaptations (Reed et al., 2015) in case they lack the same adaptative characteristics. Additional genetic studies are required to understand the risks and potential benefits of interbreeding between the resident wild stocks and the migratory stock maintained in captivity and used for stockings and enhancement projects in the region.

Our results suggest that the existing wild populations in the region cannot be supported with the hatchery brood stock without risking the unique genetic composition in the wild populations. Thus, the management decisions on the wild brown trout populations need to balance the low diversity and *N*_e_ indicating a high extinction risk with the conservation of seemingly unique genetic composition. Particularly, large enough unique populations should not be mixed with hatchery stocking in line with the concept of Evolutionarily Significant Units proposed by Fraser and Bernatchez (2001), while the extremely small populations might benefit from the increase of genetic variation from controlled stocking with regional strains.

### 4.3 Recommendations for conservation genetics studies

The resolution power of the marker depends on the number and frequency of alleles and loci available for each population. Microsatellites can usually carry a several‐fold higher number of alleles than SNPs, but the number of loci can be increased enormously in RADseq analysis compared to microsatellites. Thus, the abundance of SNP loci creates an inevitable advantage in resolution power, especially in cases where microsatellites may not function optimally due to high relatedness within populations or population bottlenecks. Consequently, although microsatellites have been central for characterizing post‐glacial phylogenetic relationships in salmonids (e.g., Hansen, Mensberg, & Berg, 1999; Koskinen, Knizhin, Primmer, Schlotterer, & Weiss, 2002; Säisä et al., 2005), and many studies report comparable estimates of genetic divergence between microsatellite and SNP markers, RADseq approach could be preferred over microsatellites for detailed phylogeography studies.

Our study indicates the benefits or RADseq can also be pronounced for small populations with limited genetic diversity due to prior population bottlenecks, which is often the case for endangered populations. Thus, the choice of marker to be used in future studies depends on the goals of the study as well as available resources; compared to microsatellite analysis, RADseq still carries higher per‐individual costs, although the overall price is decreasing with decreasing sequencing and reagent costs. There is a relatively high labor and time cost associated with establishing a new microsatellite panel, which RADseq circumvents by having similar preparation costs regardless of prior knowledge on markers. For projects aiming solely at describing population divergence across landscapes using a multiallelic microsatellites panel previously developed for the focal species, the benefits of switching to a RADseq approach can be limited. In contrast, many detailed questions on relatedness, demography, selection, genetic diversity or fine‐scale population divergence greatly benefit from an approach based on a large number of SNPs (Andrews et al., 2016). The required number of SNPs for these questions, and therefore sequencing depth and cost, varies by species and populations, but is likely lower for relatedness analysis than for, e.g., HFC studies. In conclusion, SNPs were more informative than microsatellites for describing relatedness and had much higher resolution with low sample sizes from small and isolated populations.

## CONFLICT OF INTEREST

The authors declare no conflict of interest.

## AUTHOR CONTRIBUTIONS

Conceptualization: AVai, SUH, AL, JMP. Investigation: AL, MLK, JK, JMP. Formal analysis: AL, JMP. Writing –Original draft: JMP, AL. Writing –Review and editing: JMP, AL, SUH, AVas, PH, AH, MLK, JK, AVai. Visualization: JMP, AL. Supervision: AVai, SUH, AVas. Funding acquisition: AVai, AH, PH.

## Data Availability

All sequence data have been deposited in the NCBI Sequence Read Archive (accession number SRP125540). All raw microsatellite data are available in Figshare (https://doi.org/10.6084/m9.figshare.5314666).
