# Supplementary figures and images for: Comparing RADseq and microsatellites for estimating genetic diversity and relatedness — Implications for brown trout conservation
Source: Ecol Evol. 2019 Feb 6;9(4):2106–20. doi: 10.1002/ece3.4905 (PMC6392366; doi:10.1002/ece3.4905)

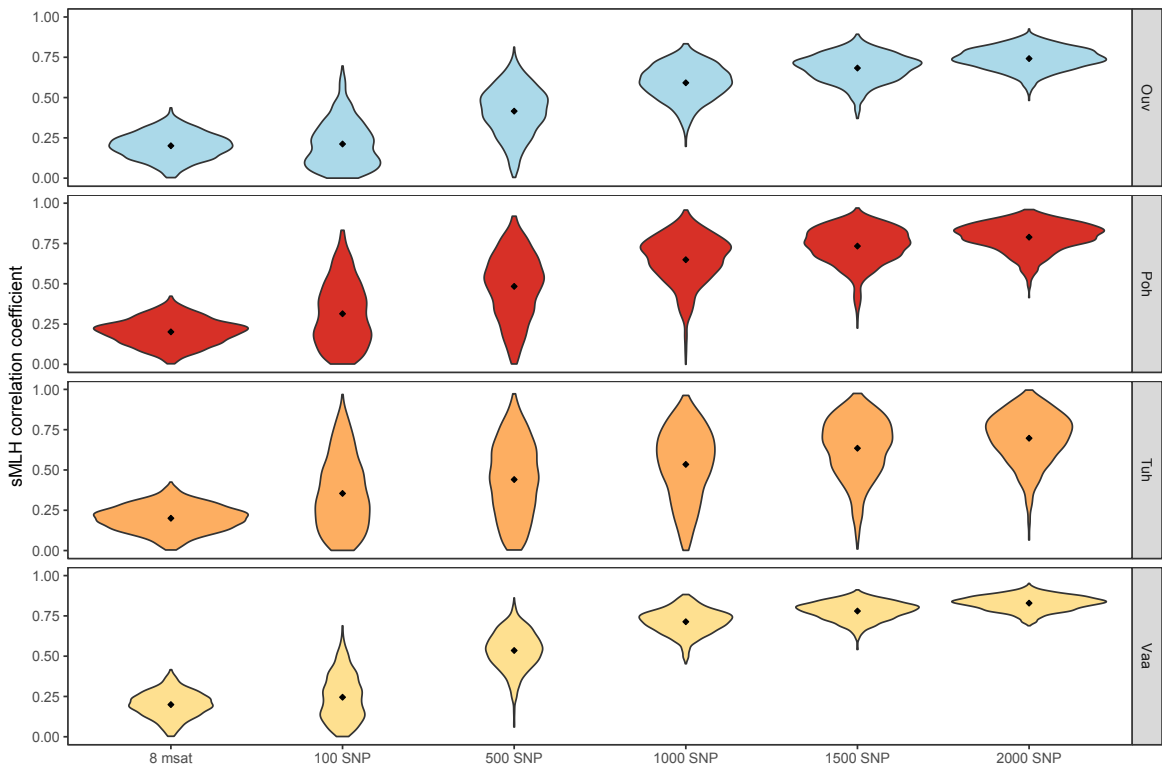

Supplement: Supplementary file 1 [file ECE3-9-2106-s001.pdf]
