# Supplementary material for: Comparing RADseq and microsatellites for estimating genetic diversity and relatedness — Implications for brown trout conservation
Source: Ecol Evol. 2019 Feb 6;9(4):2106–20. doi: 10.1002/ece3.4905 (PMC6392366; doi:10.1002/ece3.4905)
Supplement: Supplementary file 2 [file ECE3-9-2106-s002.docx]

|  | Locus | Reference | Multiplex | Dye | Primer  concentration |
| --- | --- | --- | --- | --- | --- |
| 1 | *BS131* | Estoup et al. 1998 | MP 1 | VIC | 0.03 µM |
| 2 | *OneU9* | Scribner et al. 1996 | MP 2 | VIC | 0.03 µM |
| 3 | *SSa197* | O’reilly et al. 1996 | MP 1 | NED | 0.02 µM |
| 4 | *SSa289* | McConnell et al. 1995 | MP 1 | PET | 0.30 µM |
| 5 | *Ssa407* | Cairney et al. 2000 | MP 1 | NED | 0.15 µM |
| 6 | *SSa85* | McConnell et al. 1995 | MP 2 | VIC | 0.02 µM |
| 7 | *Ssosl311* | Slettan et al. 1995 | MP 2 | NED | 0.07 µM |
| 8 | *SSosl417* | Slettan et al. 1995 | MP 1 | PET | 0.04 µM |
| 9 | *SSosl438* | Slettan et al. 1996 | MP 2 | VIC | 0.07 µM |
| 10 | *SSsp1605* | Paterson et al. 2004 | MP 2 | NED | 0.04 µM |
| 11 | *SSsp2201* | Patterson et al. 2004 | MP 1 | 6-FAM | 0.03 µM |
| 12 | *Str15INRA* | Estoup et al. 1993 | MP 1 | 6-FAM | 0.05 µM |
| 13 | *Str60lNRA* | Estoup et al. 1993 | MP 2 | PET | 0.04 µM |
| 14 | *Str73lNRA* | Estoup et al. 1993 | MP 1 | VIC | 0.04 µM |
| 15 | *Str85lNRA* | Presa & Guyomard 1996 | MP 2 | 6-FAM | 0.40 µM |
| 16 | *Strutt58* | Poteaux et al. 1999 | MP 2 | 6-FAM | 0.30 µM |

Table S1. Details of microsatellite loci, multiplexes, dyes and primer concentrations used in the analysis.

Table S2. A summary of read counts and barcodes for RADseq samples. Samples excluded for missing data or used for measuring error rate indicated in Info.

| Barcode | Sample | Raw reads | Low quality reads | Retained reads | Population | Info |
| --- | --- | --- | --- | --- | --- | --- |
| TCACTGCAG | 1 | 2628954 | 21473 | 2607481 | Pohjajoki |  |
| GTAATGCAG | 2 | 1958902 | 15908 | 1942994 | Pohjajoki |  |
| ACTATGCAG | 3 | 150203 | 1989 | 148214 | Pohjajoki |  |
| AGGCTGCAG | 4 | 1481577 | 10520 | 1471057 | Pohjajoki |  |
| GGTGTTGCAG | 7 | 1742238 | 13507 | 1728731 | Pohjajoki |  |
| CAGATGCAG | 11 | 1722265 | 13574 | 1708691 | Pohjajoki |  |
| ACAAATGCAG | 12 | 1494508 | 11290 | 1483218 | Pohjajoki |  |
| CTAGCTGCAG | 13 | 2480148 | 20877 | 2459271 | Pohjajoki |  |
| CCAGCTTGCAG | 18 | 2191678 | 17179 | 2174499 | Pohjajoki |  |
| CTGTATGCAG | 26 | 1557906 | 14831 | 1543075 | Pohjajoki | excluded |
| CATCGTTGCAG | 27 | 2102156 | 16950 | 2085206 | Pohjajoki |  |
| GAGGATGCAG | 28 | 1923016 | 14235 | 1908781 | Pohjajoki |  |
| CTTCCATGCAG | 34 | 361751 | 3630 | 358121 | Tuhkajoki | excluded |
| CGATTGCAG | 38 | 2125419 | 17548 | 2107871 | Tuhkajoki |  |
| TGCATGCAG | 41 | 43024 | 388 | 42636 | Tuhkajoki | excluded |
| CCTACTGCAG | 42 | 2015701 | 17146 | 1998555 | Tuhkajoki |  |
| CCACAATGCAG | 43 | 2653964 | 21624 | 2632340 | Tuhkajoki |  |
| GTACTTTGCAG | 44 | 1980250 | 16357 | 1963893 | Tuhkajoki |  |
| AACGCCTTGCAG | 45 | 1658982 | 10840 | 1648142 | Tuhkajoki |  |
| GGAAGATGCAG | 50 | 2784771 | 23085 | 2761686 | Tuhkajoki |  |
| TCTGTGATGCAG | 51 | 1962770 | 14668 | 1948102 | Tuhkajoki |  |
| TACATTGCAG | 52 | 1411619 | 11978 | 1399641 | Tuhkajoki |  |
| ACGTGGTATGCAG | 56 | 536914 | 3627 | 533287 | Tuhkajoki |  |
| TGCGATGCAG | 61 | 2637840 | 20480 | 2617360 | Vaarainjoki |  |
| GTCGATTTGCAG | 62 | 1981699 | 15509 | 1966190 | Vaarainjoki |  |
| GTATTTGCAG | 64 | 1376896 | 11933 | 1364963 | Vaarainjoki |  |
| AACTTGCAG | 65 | 1037351 | 7716 | 1029635 | Vaarainjoki |  |
| ACCTAATGCAG | 66 | 1148370 | 8473 | 1139897 | Vaarainjoki |  |
| AAAAGTTTGCAG | 67 | 622385 | 4790 | 617595 | Vaarainjoki |  |
| TTCTCTGCAG | 68 | 2143671 | 19612 | 2124059 | Vaarainjoki |  |
| ACCGTTGCAG | 69 | 1407994 | 9786 | 1398208 | Vaarainjoki |  |
| AGGATTGCAG | 70 | 1385373 | 10285 | 1375088 | Vaarainjoki |  |
| TCACCTGCAG | 71 | 1285265 | 11096 | 1274169 | Vaarainjoki |  |
| TCGAAGATGCAG | 72 | 822717 | 6501 | 816216 | Vaarainjoki |  |
| ACAGGGAATGCAG | 73 | 456433 | 2905 | 453528 | Vaarainjoki |  |
| CGGTAGATGCAG | 74 | 546131 | 4342 | 541789 | Vaarainjoki |  |
| GCGTTGCAG | 75 | 883485 | 7484 | 876001 | Vaarainjoki |  |
| AGCCCTGCAG | 76 | 1518393 | 12004 | 1506389 | Vaarainjoki |  |
| TCGTTTGCAG | 77 | 897751 | 7452 | 890299 | Vaarainjoki |  |
| CATCTTGCAG | 78 | 1959195 | 16043 | 1943152 | Vaarainjoki |  |
| TCTCAGTCTGCAG | 79 | 1323152 | 10389 | 1312763 | Vaarainjoki |  |
| GCTCTATGCAG | 80 | 901529 | 7590 | 893939 | Vaarainjoki |  |
| GGAACTGCAG | 81 | 1729648 | 14754 | 1714894 | Vaarainjoki |  |
| GTCAATGCAG | 82 | 697217 | 5860 | 691357 | Vaarainjoki |  |
| GCCAGTTGCAG | 83 | 1390430 | 11758 | 1378672 | Vaarainjoki |  |
| TAATATGCAG | 84 | 1660721 | 14223 | 1646498 | Vaarainjoki |  |
| GGACCTATGCAG | 85 | 1433560 | 12032 | 1421528 | Vaarainjoki |  |
| GCTGTGGATGCAG | 86 | 510489 | 3696 | 506793 | Vaarainjoki |  |
| TTCAGATGCAG | 87 | 622889 | 5218 | 617671 | Vaarainjoki |  |
| AGTGGATGCAG | 88 | 922148 | 5808 | 916340 | Vaarainjoki |  |
| TGGCTATGCAG | 89 | 547658 | 4610 | 543048 | Vaarainjoki |  |
| GAGATATGCAG | 90 | 1503856 | 11968 | 1491888 | Hatchery |  |
| ATGCCTTGCAG | 91 | 860431 | 6239 | 854192 | Hatchery |  |
| GTTGAATGCAG | 92 | 860677 | 6233 | 854444 | Hatchery |  |
| ATATGTTGCAG | 94 | 1124170 | 8820 | 1115350 | Hatchery | excluded |
| ATTAATTTGCAG | 95 | 1655349 | 12226 | 1643123 | Hatchery |  |
| TATTTTTTGCAG | 96 | 868618 | 7307 | 861311 | Hatchery |  |
| AATATGCTGCAG | 97 | 1058249 | 7829 | 1050420 | Hatchery |  |
| GATCTGCAG | 98 | 3141519 | 24678 | 3116841 | Hatchery |  |
| TAACGATGCAG | 99 | 2015398 | 16487 | 1998911 | Hatchery |  |
| TAGCGGATGCAG | 100 | 1457281 | 11692 | 1445589 | Hatchery |  |
| TGCTGGATGCAG | 101 | 917287 | 7846 | 909441 | Hatchery |  |
| CTCCTGCAG | 102 | 1410113 | 11861 | 1398252 | Hatchery |  |
| CTTGCTTTGCAG | 104 | 1366525 | 11518 | 1355007 | Hatchery |  |
| GCTTATGCAG | 105 | 1790860 | 15946 | 1774914 | Hatchery |  |
| ACGTGTTTGCAG | 106 | 1321434 | 9323 | 1312111 | Hatchery |  |
| CATAAGTTGCAG | 107 | 1387199 | 11259 | 1375940 | Hatchery |  |
| CGCTTTGCAG | 108 | 2480213 | 23453 | 2456760 | Hatchery |  |
| ATTGATGCAG | 109 | 1508358 | 9968 | 1498390 | Hatchery |  |
| TAGCATGCTGCAG | 110 | 928908 | 7143 | 921765 | Hatchery |  |
| AACCGAGATGCAG | 111 | 1446308 | 10867 | 1435441 | Hatchery |  |
| ATTGGATTGCAG | 112 | 1227619 | 7842 | 1219777 | Hatchery |  |
| TAGGCCATTGCAG | 113 | 1166336 | 8732 | 1157604 | Hatchery |  |
| TGCAAGGATGCAG | 114 | 1797588 | 14840 | 1782748 | Hatchery |  |
| GGATTGGTTGCAG | 115 | 1090744 | 9385 | 1081359 | Hatchery |  |
| TAGGAATGCAG | 116 | 1905245 | 13501 | 1891744 | Hatchery |  |
| ACGACTACTGCAG | 117 | 1348136 | 9806 | 1338330 | Hatchery |  |
| CCATGGGTTGCAG | 118 | 1735468 | 14790 | 1720678 | Hatchery |  |
| CGTGTGGTTGCAG | 119 | 1415037 | 10931 | 1404106 | Hatchery | excluded |
| CGCGGAGATGCAG | 120 | 1195643 | 9839 | 1185804 | Hatchery |  |
| ATCGTATGCAG | 157 | 684710 | 4545 | 680165 |  | seq error control |
| CGCGGTTGCAG | 172 | 802890 | 6675 | 796215 |  | seq error control |
| CTATTATGCAG | 196 | 1596510 | 14204 | 1582306 |  | seq error control |
| GGTTGTTGCAG | 282 | 964080 | 8056 | 956024 |  | seq error control |
| GAACTTCTGCAG | 301 | 1258246 | 10680 | 1247566 |  | seq error control |
| GCGGAATTGCAG | 422 | 825277 | 6010 | 819267 |  | seq error control |
